# Synergistic Valorization of Ternary Industrial Solid Wastes for Sustainable Loess Stabilization: Mix Optimization, Strength Evolution, and Microstructural Mechanisms

**DOI:** 10.3390/ma19183999

**Published:** 2026-09-20

**Authors:** Anhua Xu, Jiahong Li, Yushu Jing, Yonghai Gu, Yuanji Li, Yindong Xu, Bowen Guan

**Affiliations:** 1 School of Water Resources and Civil Engineering, Qinghai Polytechnic University, Xining 810016, China; yuanjili5201@163.com (Y.L.); x04980217@163.com (Y.X.); 2 Qinghai Provincial Key Laboratory of Tibet Plateau Highway Construction and Maintenance Technology, Xining 810003, China; 3 School of Materials Science and Engineering, Chang’an University, Xi’an 710061, China; 2024131074@chd.edu.cn (J.L.); ysjing@chd.edu.cn (Y.J.); 4 Qinghai Xingli Highway & Bridge Engineering Co., Ltd., Xining 810002, China; guyonghai2016@163.com

**Keywords:** fly ash, lithium slag, magnesium slag, stabilized loess, strength formation mechanism, response surface methodology

## Abstract

This study investigates the mechanical properties and strength formation mechanism of loess solidified with a ternary blend of fly ash, lithium slag, and magnesium slag. The mix proportion was optimized using response surface methodology with a Box–Behnken design. Unconfined compressive strength (UCS) tests, digital image correlation (DIC), X-ray diffraction (XRD), and scanning electron microscopy (SEM) were employed for evaluation. The optimal 7-day mix (15.926 wt.% fly ash, 10.158 wt.% lithium slag, and 6.427 wt.% magnesium slag) achieved a UCS of 0.974 MPa, while the 28-day optimum (16.064 wt.% fly ash, 10 wt.% lithium slag, and 2 wt.% magnesium slag) yielded 1.834 MPa. Strength development followed a quadratic nonlinear model at early age, shifting to a linear superposition model at 28 days. XRD and SEM revealed that strength enhancement originates from synergistic pozzolanic and hydration reactions under alkaline activation, producing C-S-H gel and ettringite (AFt) that fill pores and cement soil particles. The ternary system demonstrates effective utilization of industrial solid wastes for loess stabilization.

## 1. Introduction

Loess covers over 60% of Northwest China’s land area and provides the foundational layer for critical infrastructure projects such as highways, railways, and building foundations. Moreover, the loess in Northwest China may suggest that significant engineering deficiencies are inherent, including a high void ratio, weak interparticle cementation, and pronounced collapsibility. In light of the combined effects of water infiltration and external loading, the loess structure could indicate a high susceptibility to failure, which may trigger engineering hazards such as subgrade settlement, slope instability, and differential foundation deformation. Furthermore, the evidence could demonstrate that these issues pose severe threats to the construction quality and operational safety of infrastructure projects [1]. However, the findings may suggest that conventional loess stabilization methods predominantly rely on materials such as cement and lime, though these materials exhibit significant limitations despite their capacity to enhance mechanical properties to a certain extent. Given that cement production demonstrates high energy consumption and substantial carbon emissions, the results could indicate that this conflicts with the dual carbon goals of peak carbon emissions and carbon neutrality. Notwithstanding the slow strength development observed, the evidence may suggest that lime stabilization suffers from poor water stability as well. Thus, the key challenge could be that aligning with the dual demands of large-scale infrastructure construction and sustainable green development in Northwest China appears increasingly difficult [2,3].

Against this backdrop, the development of alternative stabilizing materials that are low-cost, low-pollution, and widely available may suggest that this has become a prominent research focus in the field. However, the significant findings could indicate that numerous domestic researchers have conducted extensive studies on loess improvement and performance enhancement. Thus, the results may demonstrate that Zhang et al. [4] focused on overly wet loess and proposed four improvement schemes: single addition of fly ash, alkali-activated fly ash, slag–fly ash, and alkali-activated slag–fly ash. In light of the comparative experimental verification, the evidence could demonstrate that the alkali-activated slag–fly ash-modified loess exhibited the lowest moisture content, superior gradation, maximum shear strength, and favorable resistance to compressive deformation, establishing it as the optimal choice among the four schemes. He et al. [5] utilized a TSZ-3 strain-controlled triaxial shear apparatus to conduct important tests on fly ash-lime-stabilized loess with varying moisture contents and compaction degrees under different confining pressures. Furthermore, the key findings may suggest that the stress–strain curves of the stabilized loess predominantly exhibited strain-softening behavior, characterized by brittle failure. Notwithstanding the significant influence on cohesion, the evidence could indicate that compaction degree and moisture content exerted relatively minor effects on the internal friction angle of the stabilized loess. Therefore, the results may demonstrate that Zhou et al. [6] systematically investigated the influence of delay time on the mechanical properties of fly ash-stabilized loess used as subgrade fill. Additionally, the significant findings could indicate that through compaction tests, unconfined compressive strength tests, and direct shear tests conducted at different delay times, the results appear to demonstrate that the overall structural looseness of the stabilized soil gradually intensified, with the original large, aggregated particle structure progressively disintegrating into a dispersed granular state.

China generates an enormous volume of industrial solid waste annually, and the evidence may suggest that the stockpiling and disposal of fly ash, lithium slag, and magnesium slag not only occupy extensive land resources but also pose significant risks of soil and water contamination. Moreover, the findings could demonstrate that their resource utilization appears to be an urgent imperative [7,8,9,10]. Furthermore, the combined application of these three industrial solid wastes in loess stabilization may suggest that substantial synergistic potential exists, offering a novel approach to overcoming the important limitations inherent in conventional stabilization technologies. Given that the evidence demonstrates significant environmental concern, the results could indicate that this approach holds key value for the field.

Fly ash is the primary solid waste by-product of coal-fired thermal power plants, and the evidence may suggest that its massive accumulation not only causes environmental pollution and resource wastage but also entails considerable carbon emission risks [11]. However, the significant findings could demonstrate that this material is characterized by wide availability, low cost, and an abundance of reactive components such as SiO_2_ and Al_2_O_3_, which impart latent pozzolanic activity. In light of these important reactive properties, the results may indicate that under specific conditions, fly ash can react with water to form cementitious products capable of effectively filling loess voids and cementing soil particles [12]. Therefore, the evidence could suggest that lithium slag, a waste generated during lithium salt production, similarly contains substantial amounts of reactive SiO_2_ and Al_2_O_3_, along with minor quantities of Li_2_O. Moreover, the key findings may suggest that under alkaline activation, lithium slag could accelerate the hydration process and synergistically interact with fly ash to enhance the strength of the stabilized matrix [13]. Furthermore, the significant evidence could demonstrate that magnesium slag, a by-product of metallic magnesium smelting with approximately 5–6 tons of slag generated for every ton of magnesium produced, is rich in alkaline components such as CaO and MgO. Given that the evidence demonstrates that upon hydration this material establishes an alkaline environment, the results may indicate that it effectively activates the reactivity of both fly ash and lithium slag while concurrently optimizing the ionic environment of the loess [14,15,16,17]. Additionally, the findings could suggest that this process appears to provide favorable conditions for the strength development of the stabilized body. Results show a favorable environment.

The combined incorporation of these three industrial solid wastes for loess stabilization may suggest that it serves a dual purpose, facilitating the efficient consumption and valorization of industrial solid waste in alignment with green development principles. However, the significant evidence could demonstrate that it also enables the substitution of conventional stabilizing materials, thereby reducing important engineering costs. The results may indicate that this approach possesses both significant environmental value and demonstrated research feasibility [18,19,20]. Moreover, the evidence could suggest that it appears to elucidate the core logic underpinning the resource utilization of solid waste materials. However, the significant findings may demonstrate that understanding this dual value could indicate broader implications for the field. In light of these results, the evidence could suggest that the approach merits further systematic investigation. The approach shows dual value.

Although previous studies have demonstrated the effectiveness of industrial solid wastes in improving the mechanical properties of loess, most existing research has focused on binary solid waste systems, such as fly ash–slag, fly ash–lime, and other binary combinations [4,5,6]. In contrast, the synergistic effects of ternary solid waste systems consisting of fly ash, lithium slag, and magnesium slag remain insufficiently understood. In particular, systematic investigations into the optimization of component proportions, strength evolution at different curing ages, and the underlying mechanisms governing microstructural development are still limited. Furthermore, the role of magnesium slag in providing an alkaline environment to activate the reactivity of fly ash and lithium slag, as well as the relationship between hydration products, microstructural evolution, and macroscopic strength enhancement, requires further clarification [11,12,13,14,15,16,17].

Therefore, this study aims to develop a ternary industrial solid waste-based stabilization system for loess using fly ash, lithium slag, and magnesium slag. Response surface methodology (RSM) was employed to optimize the mixture proportions, while unconfined compressive strength (UCS) tests were conducted to evaluate the mechanical performance under different curing conditions. In addition, digital image correlation (DIC), X-ray diffraction (XRD), and scanning electron microscopy (SEM) analyses were integrated to reveal the deformation characteristics, phase evolution, and microstructural mechanisms responsible for strength development. This study provides new insights into the synergistic utilization of industrial solid wastes and offers a sustainable approach for improving loess stabilization performance [21,22].

## 2. Experimental Section

### 2.1. Raw Material Composition

#### 2.1.1. Raw Materials

**Figure 1:**
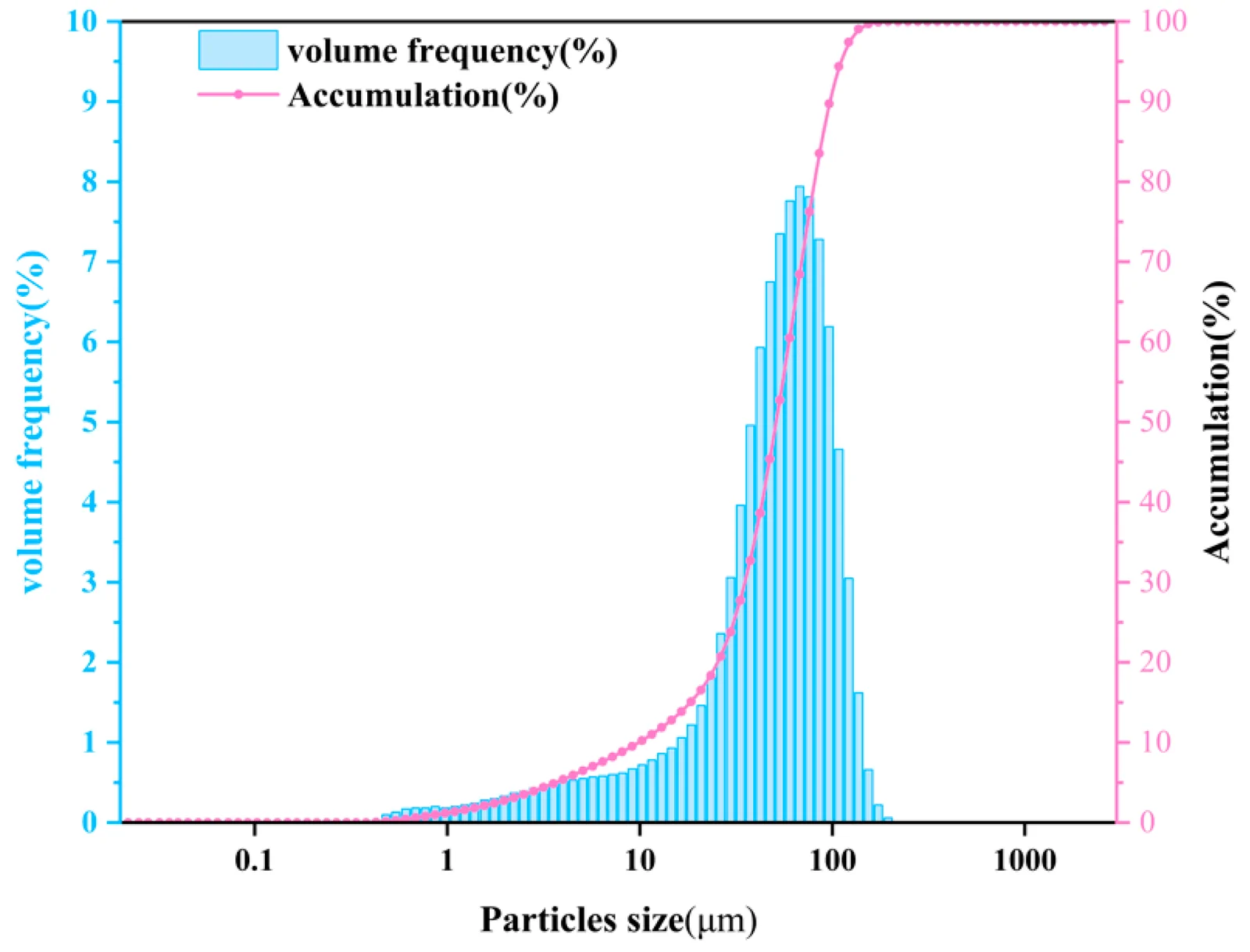
Particle size distribution curve of the loess.

The loess used in this experiment was sourced from Qinghai Province, China. However, the soil samples may suggest that the preparation method could have a significant influence on the results, as the evidence indicates that the samples were crushed. Moreover, the significant findings could indicate that the particle size distribution of the loess appears to reflect key characteristics of the material. In light of the results, the study may suggest that the distribution curve demonstrates important patterns, as determined using a laser particle size analyzer (Bettersize 2600, Bettersize Instruments Ltd., Dandong, China). Findings show the curve presented in Figure 1.

#### 2.1.2. Physical Properties of the Loess

The physical properties of the loess material used as subgrade fill in this experiment may suggest that laboratory evaluation could indicate several important characteristics. However, the findings from the particle size analysis, Atterberg limits testing, and light compaction testing appear to demonstrate that the evidence supports a comprehensive assessment of the test soil. Furthermore, the significant results derived from these procedures could indicate that the basic physical property indices align with established standards. In view of the results, Table 1 summarizes the indices determined via the Test Methods of Soils for Highway Engineering (JTG 3430-2020 [23]). The data show that these indices meet the standard requirements.

#### 2.1.3. Characteristics of Stabilizing Materials

**Figure 2:**
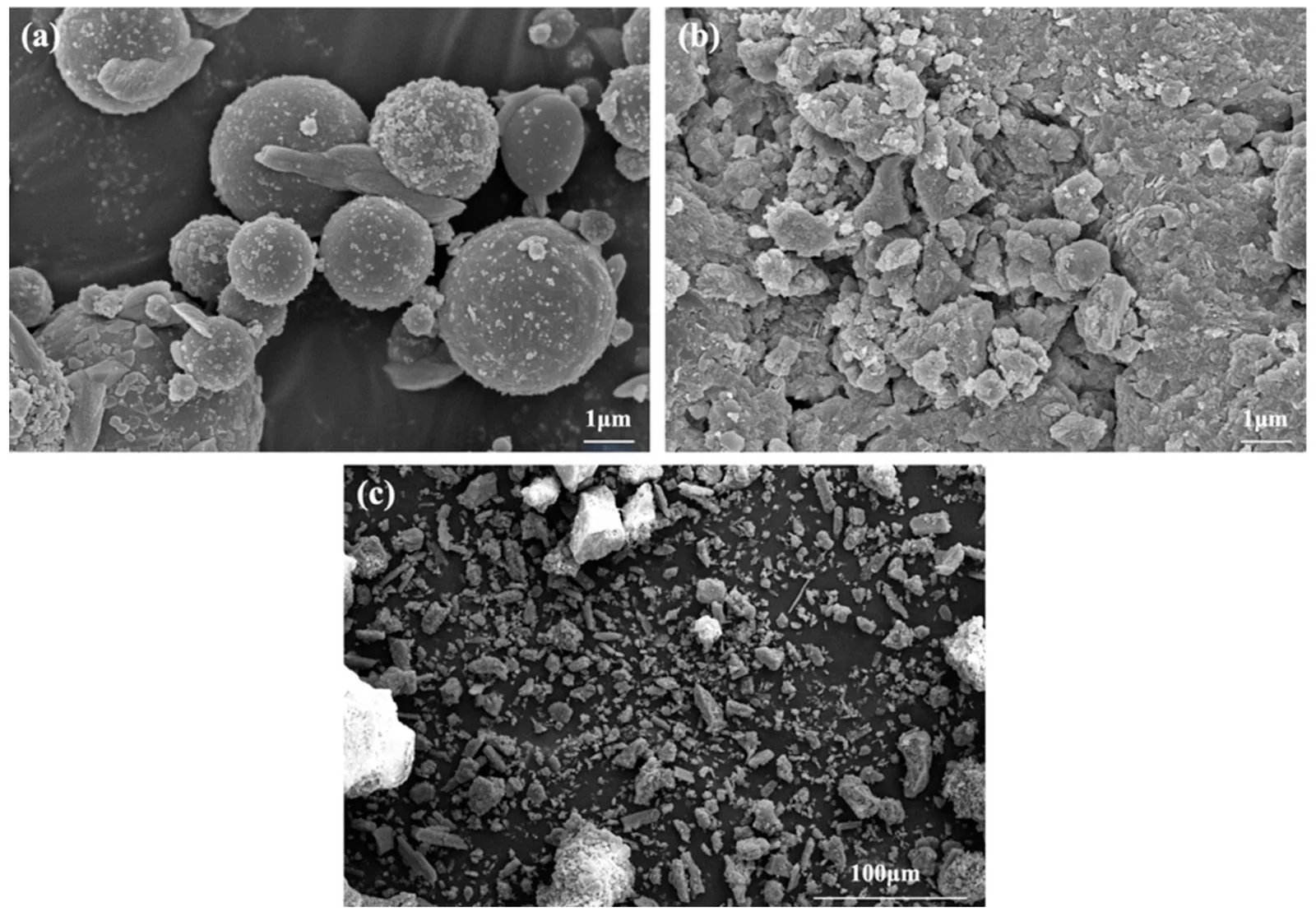
SEM images of (**a**) fly ash, (**b**) magnesium slag, and (**c**) lithium slag.

**Figure 3:**
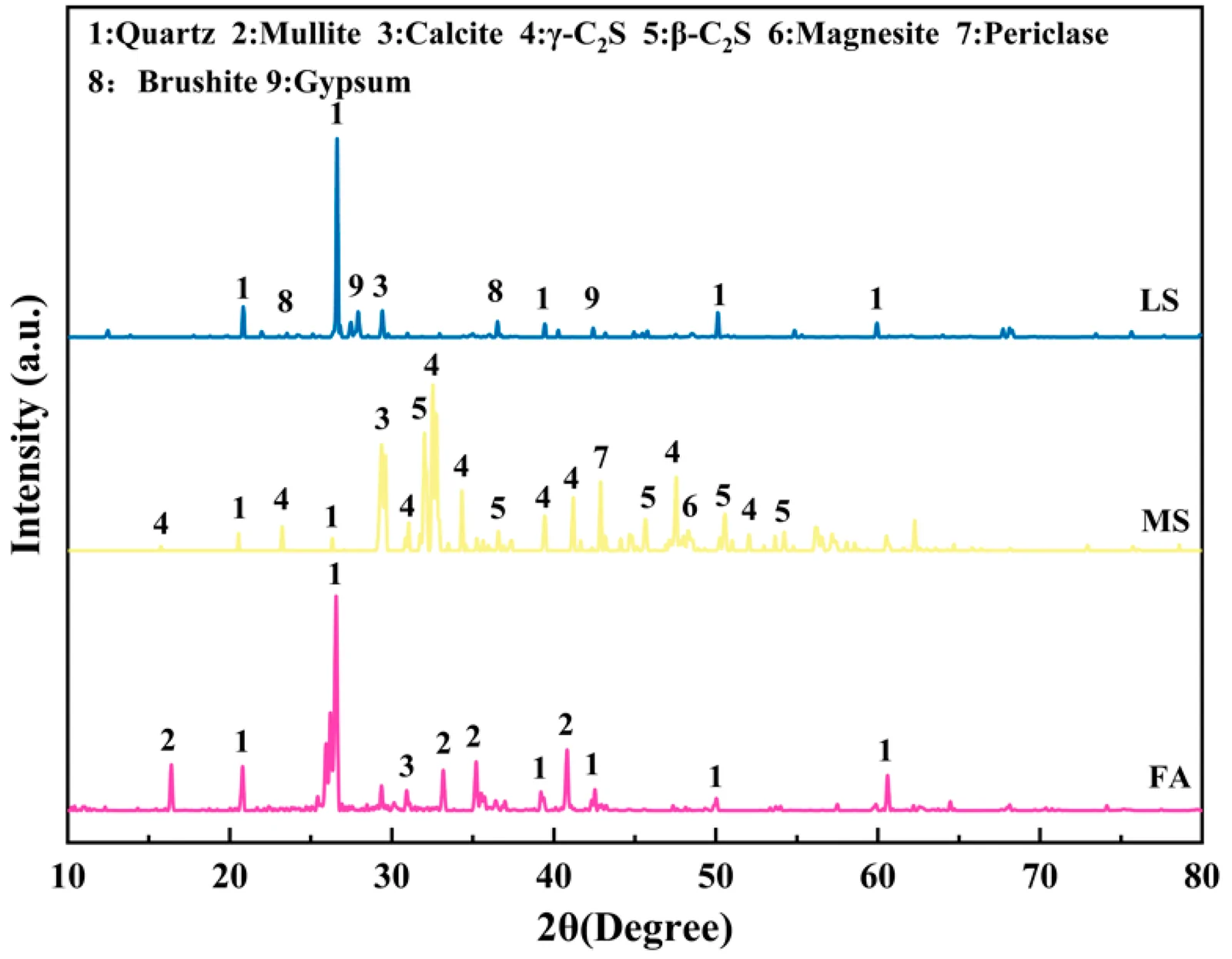
XRD patterns of raw materials.

Figure 2 and Figure 3 have shown the microscopic morphological features and mineralogical phase compositions of the three raw materials used in this work: fly ash (FA), magnesium slag (MS), and lithium slag (LS). Furthermore, based on the combined results of the SEM images and XRD patterns, it is likely that fly ash is mainly composed of spherical particles with different sizes and smooth surfaces and latent pozzolanic activity, and the existence of these spherical particles indicates that dense coverage with numerous spherical glassy microspheres appears significantly, while the amount of irregular agglomerates also exists locally. Consequently, it is likely that the formed significant agglomerates are mainly constituted by the agglomeration of elongated acicular crystalline particles and spherical glassy microspheres; the acicular crystals could be considered as sillimanite, Al_2_O_3_·SiO_2_, or mullite, 3Al_2_O_3_·2SiO_2_. Regarding the exhibited clustering behavior, it seems that the formed resultant structural arrangements are relevant to reactivity. Magnesium slag is composed of rough and irregular blocky particles with porous surfaces. It seems that the rough surface morphology may be responsible for the enhanced physical interlocking and mechanical bonding, and, consequently, the corresponding XRD pattern could suggest that the mineralogical composition of magnesium slag seems to appear relatively complex, while the diffraction peaks of γ-C_2_S and β-C_2_S phases with potential hydration or carbonation reactivity are dominant. In addition to the dominant phases, significant evidence indicates that periclase, magnesite and calcite phases appear as existing. Notwithstanding the above complex phase compositions, it seems that the key data could suggest that they are likely to exhibit important implications for reactivity behavior. The evidence found that the loess microstructure appears loose and porous. Consequently, the evidence indicated that the formed resultant particles could likely appear mainly aggregated in platy stacks or agglomerated in irregular aggregates, while the interparticle voids are distinctly developed. Regarding the significant outcomes above, the primary composition of high-strength quartz and calcium-bearing calcite could likely appear as existing in the crystalline phases. Moreover, the available data indicate that such phases are characteristic of the loess material’s sedimentary origin. Fly ash (FA) was sourced from a coal-fired thermal power plant in Shaanxi Province, China. Lithium slag (LS), a by-product generated during lithium carbonate production, was obtained from a lithium chemical plant in Jiangxi Province, China. Magnesium slag (MS), a by-product from the magnesium smelting industry, was provided by a magnesium plant in the Ningxia Hui Autonomous Region, China. The chemical compositions and physical properties of these materials are presented in Table 2. FA is abundant in SiO_2_ and Al_2_O_3_, providing a reactive source of aluminosilicates. LS contains substantial amounts of SiO_2_, Al_2_O_3_, and CaO, which confer pozzolanic reactivity. MS features high CaO and MgO contents, which increase the alkalinity of the binder system and facilitate the formation of hydration products.

The alkaline activator employed in this experiment was a sodium hydroxide (NaOH) solution, which indicates that the primary function was to provide alkaline activation for the stabilization reactions. Additionally, key results from initial experiments showed that a NaOH solution with a concentration of 5 wt.% was used for this purpose, with the total amount of solution added being dictated by the optimum moisture content requirement of the mixture. The amount of solution was determined according to the optimum moisture content (13.4%), corresponding to a liquid-to-solid ratio (L/S) of 0.112.

#### 2.1.4. Specimen Preparation

Furthermore, the specimen preparation was conducted in accordance with the Test Methods of Soils for Highway Engineering (JTG 3430-2020), which might indicate that the results could demonstrate compliance with established standards. Given that the evidence demonstrates careful material handling, the soil was air-dried naturally in a shaded, well-ventilated outdoor area for 14 days, after which the key procedures of manual homogenization, pulverization, and oven-drying were applied for subsequent use. After drying and pulverization, the soil was passed through a 2 mm sieve for subsequent specimen preparation. The sieved soil was first dry-mixed with the ternary stabilizing agent at a dosage of 20 wt.% until a visually homogeneous blend was achieved. Subsequently, the 5 wt.% aqueous NaOH solution was uniformly sprayed onto the dry mixture, in an amount required to achieve the optimum moisture content of 13.4%, and wet-mixing was conducted continuously and thoroughly to ensure an even distribution of the moisture and binder components. Furthermore, the findings may show that the mixture was put into plastic bags and left for 24 h to condition the mixture. Considering the above key findings, the cylindrical specimens Φ5 cm × H5 cm seem to show that the method of double-ended static compaction could be used to obtain a satisfactory specimen. Specimens were wrapped immediately after compaction. Notwithstanding the procedural complexity, the evidence may suggest that a thin layer of petroleum jelly was uniformly applied to the inner walls of the mold prior to compaction to facilitate demolding without causing damage. Therefore, the significant results could indicate that the compacted specimens were left undisturbed within the mold for 2 min before being gently extruded. Moreover, the key findings might demonstrate that the specimens were further wrapped in two layers of cling film after extrusion. Thus, the important evidence may suggest that the prepared specimens were placed in a curing chamber maintained at a temperature of 20 ± 2 °C and a relative humidity of 95% for the designated curing periods of 7 days and 28 days. Curing conditions support stabilization. The contents of FA, LS, and MS are all expressed as percentages by mass of the total dry soil.

### 2.2. Methodology of Experiments

#### 2.2.1. Test for Unconfined Compressive Strength

**Figure 4:**
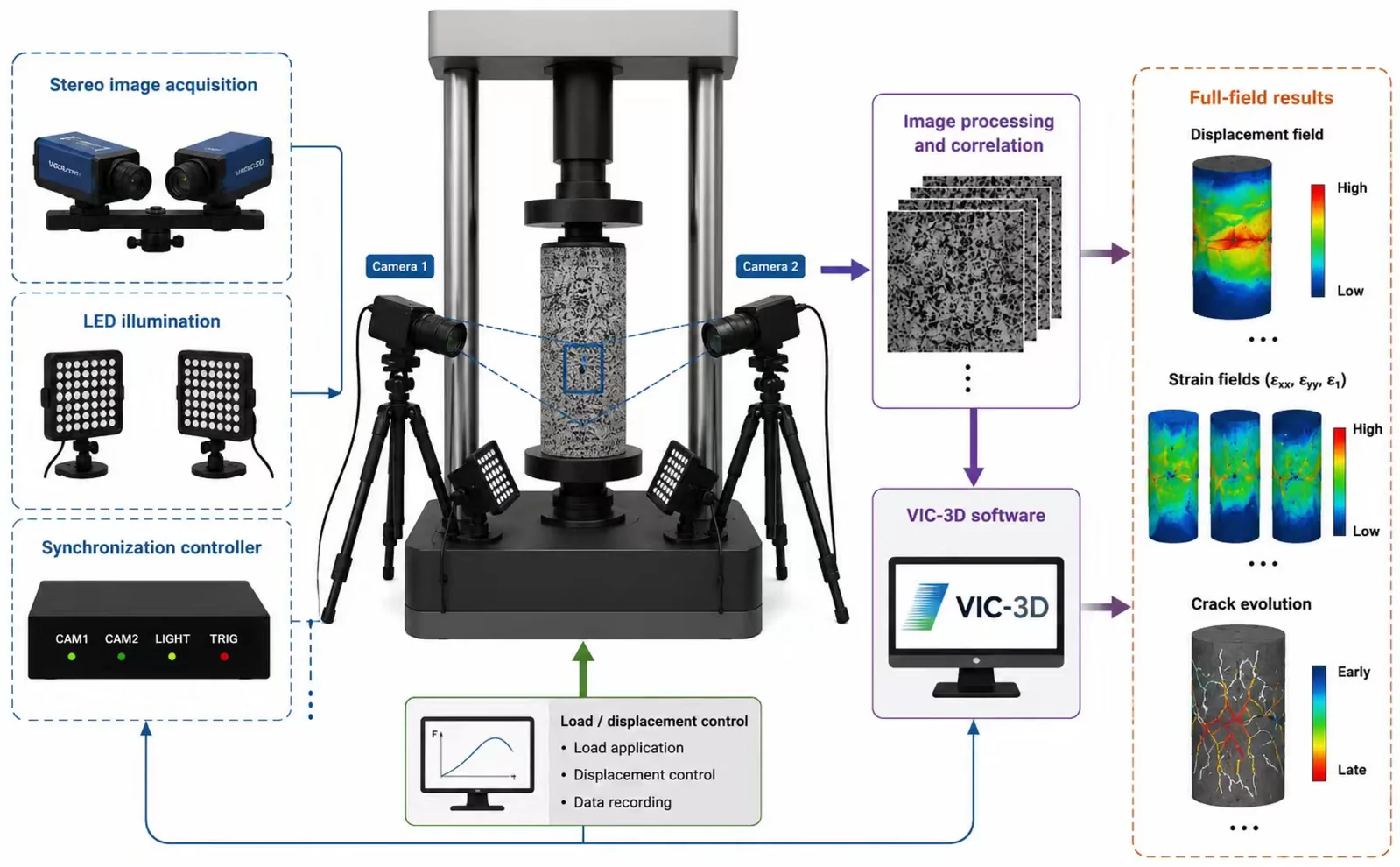
Fusion of unconfined compressive strength testing and digital image correlation.

In addition to the UCS tests, the digital image correlation (DIC) method based on the VIC-3D system (Correlated Solutions, Inc., Irmo, SC, USA) was employed to monitor the deformation evolution and failure process of specimens during unconfined compression testing [24]. The DIC system was used to obtain the full-field displacement and strain distributions on the specimen surface under different loading stages. The fusion of unconfinedcompressive strength testing and digital image correlation was shown in Figure 4.

Before testing, the camera system was calibrated using a standard calibration plate to establish the three-dimensional coordinate system and correct lens distortion. The coordinate system was established with the center of the specimen as the reference origin. Two high-resolution digital cameras were symmetrically arranged around the specimen to capture the surface deformation during loading. The image acquisition frequency was set to 5 frames per second (fps), which was sufficient to capture the progressive deformation and failure process under the relatively slow loading rate of the UCS test. Prior to DIC measurement, the specimen surface was coated with a thin layer of white paint to provide a uniform background, followed by spraying black random speckles. The speckle pattern was optimized to ensure sufficient contrast and random distribution, avoiding excessive clustering or overlapping of speckles. During image processing, the specimen surface was divided into numerous subsets, and the displacement and strain fields were calculated by tracking the grayscale changes in each subset between consecutive images. In this study, a subset size of 31 × 31 pixels and a step size of 7 pixels were adopted based on the image resolution and correlation quality. The selected parameters provided a balance between spatial resolution and correlation stability. It should be noted that an excessively small subset may reduce correlation reliability due to insufficient grayscale information, whereas an excessively large subset may reduce spatial resolution. The measurement uncertainty of the DIC system was evaluated through a static image test without mechanical loading. The calculated displacement fluctuation was less than 0.01 pixels, corresponding to a displacement measurement uncertainty of approximately ±2 μm under the current experimental configuration. The correlation coefficient during image analysis was maintained above 0.95, indicating reliable image matching quality. The DIC analysis provided the full-field displacement and strain evolution of the specimen throughout the loading process. In addition, five representative points were selected from the full-field deformation maps to quantitatively compare local deformation characteristics at different loading stages. These points were selected from typical regions of interest rather than randomly selected positions. The deformation and failure behavior were interpreted based on the combined information from the full-field strain distribution, representative point evolution, and UCS results, rather than relying solely on the five selected points.

#### 2.2.2. Microscopic Performance Testing

X-ray diffraction (XRD) serves as a valuable tool for qualitative phase identification. The underlying X-rays constitute a form of electromagnetic wave possessing extremely short wavelengths. Because of this, X-rays can pass through materials to a certain depth. It is this periodicity in the structure of crystalline materials that leads to the diffraction of the incident X-rays, thus providing accurate information about the crystal structure.

Scanning electron microscopy (SEM) was used for microstructure observation. The prepared specimen was loaded into the scanning electron microscope. A high-energy electron beam was focused and passed onto the sample surface. The sample emitted secondary electrons, which were collected by the associated detector, processed and then displayed on the fluorescent screen for the observation and photographing of the sample surface morphology.

The XRD patterns were recorded over a 2θ range of 5° to 80° at a step size of 0.02° and a scanning rate of 10°/min. Phase identification was carried out using the ICDD PDF-4+ database with the aid of Jade 6.5 software. The SEM observations were integrated with the XRD results to establish the correlations among hydration products, microstructural evolution, and macroscopic mechanical performance.

## 3. Experimental Results and Discussion

### 3.1. RSM-Guided Unconfined Compressive Strength Assessment

Response surface methodology (RSM) is mainly used to optimize the experimental process. By considering random error to the best of possible, RSM optimizes the experimental process and can minimize the number of tests while providing a reliable result. Fitting the experimental process achieved through RSM creates a continuous surface model, and through this model, reasonable ranges for each influencing factor can be determined. Compared with other methods, such as orthogonal experimental design, the continuous surface model with a continuous surface plot achieved by RSM has much better visualization performance than scattered data; thus, outcomes can be predicted at points outside the experimental domain.

In this study, RSM was used to determine the optimal proportion of the raw materials, and the 7-day and 28-day compressive strengths were chosen as the response indicators. The Box–Behnken design was implemented using Design-Expert software (Design-Expert 13.0). The magnesium slag dosage range was set at 0–13 wt.% and the lithium slag dosage range was set at 0–13 wt.%. Considering the large influence of fly ash dosage on the activity index, the fly ash dosage range was set at 0–26 wt.%.

The regression analysis encompassed linear, two-factor interaction (2FI), and quadratic models for the 7-day and 28-day compressive strengths. The goodness-of-fit of the three models relative to the two strength values is detailed in Table 3 (7-day) and Table 4 (28-day). The coefficient of determination (R^2^) was adopted as the metric for assessing fit quality and identifying the optimal regression model.

Based on the data presented in Table 3, the reduced quadratic model for the 7-day compressive strength yielded an adjusted R^2^ of 0.8512 and a predicted R^2^ of 0.6205, indicating that approximately 85.1% of the variability in the experimental data can be explained by the model. Compared with the linear and 2FI models, the reduced quadratic model provided a superior fit. It should be noted that the original full quadratic model initially exhibited a negative predicted R^2^ (–0.9904), indicating severe overfitting due to an excessive number of model terms relative to the limited sample size (*n* = 17). To address this issue, a reduced quadratic model was established by eliminating non-significant terms (AC and C^2^) via stepwise regression, which substantially improved the predictive capability. Therefore, the reduced quadratic model was adopted to fit the 7-day compressive strength.

**Figure 5:**
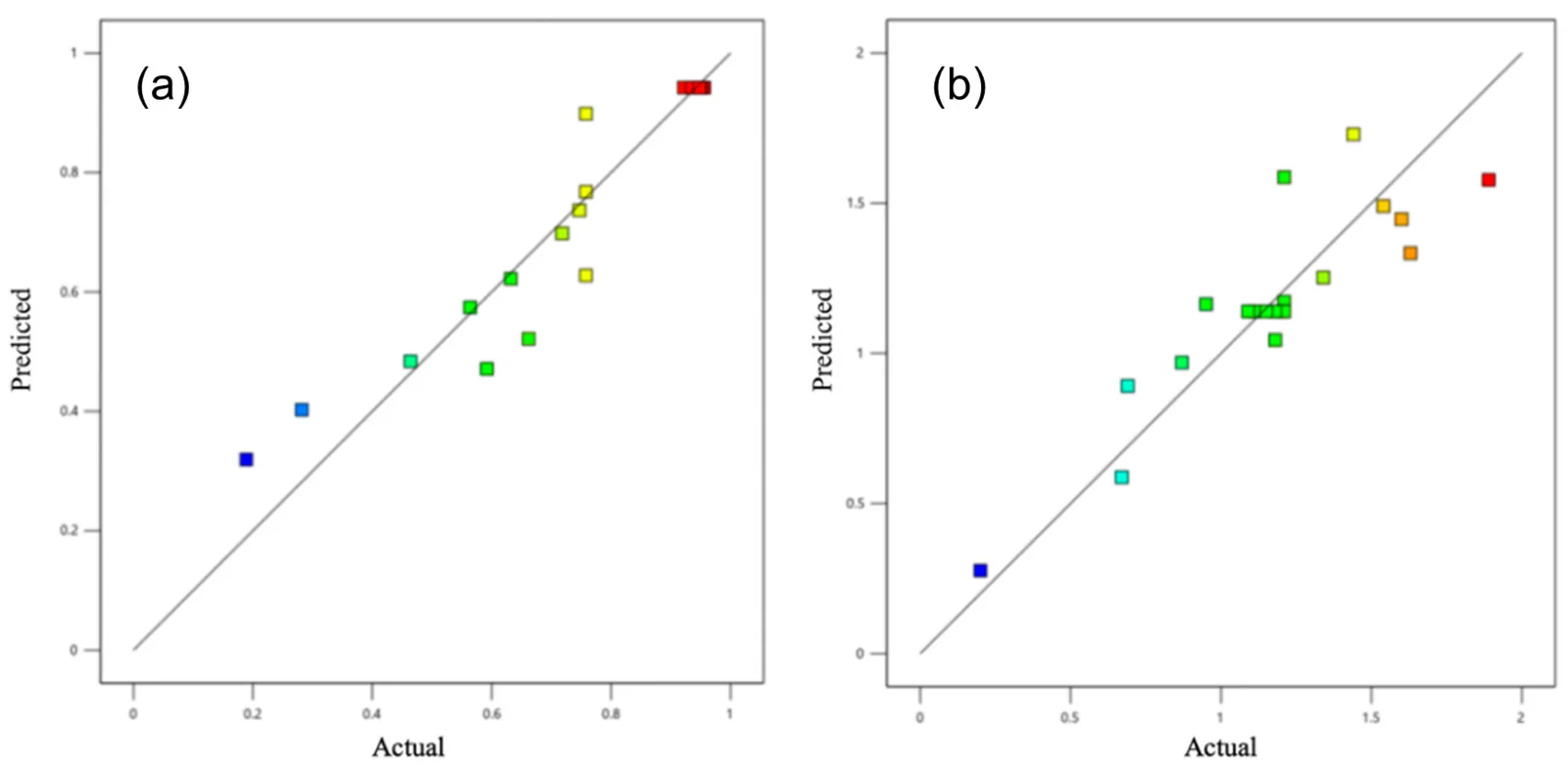
Distribution of actual versus predicted values: (**a**) 7-day curing period; (**b**) 28-day curing period.

As shown in Table 3, the lack-of-fit *p*-values for both the linear and reduced quadratic models were below 0.001, indicating statistically significant lack of fit. This suggests that the current models may not fully capture all underlying variations in the experimental data, possibly due to the limited number of experimental runs, inherent variability in the multi-component solid waste system, or the presence of higher-order interactions that could not be adequately resolved within the present design. This limitation is acknowledged, and the subsequent discussion of model predictions is therefore presented with appropriate caution. Nevertheless, the models remain useful for identifying general trends and optimal proportion ranges, as further supported by the experimental validation and microstructural evidence discussed in later sections. Distribution of actual versuspredicted values was shown in Figure 5.

The models and parameters employed in this experiment were tested at the 95% confidence level. Accordingly, a *p*-value of less than 0.05 denotes that the model and its parameters are statistically significant.

The analysis of variance (ANOVA) of influencing factors on 7-day and 28-day compressive strengths is shown in Table 5 and Table 6, respectively. ANOVA is a statistical method utilized in this study to evaluate the significance of the individual solid waste components and their interactions on the compressive strength. Within these tables, the “Source” column lists the specific model terms contributing to the variance. Here, A, B, and C denote the linear main effects of fly ash (FA), lithium slag (LS), and magnesium slag (MS), respectively; AB, AC, and BC represent their two-way interaction effects; and A^2^, B^2^, and C^2^ indicate the quadratic effects of each individual factor. As can be seen from the data in the above two tables, the *p*-values of compressive strength fitting model are all less than 0.05. Therefore, the models established by response surface methodology are statistically significant and the confidence rate is more than 95%.

Contour plots and three-dimensional response surface plots were plotted with the software to depict the relationship between compressive strength response index and pairwise interactions of influencing factors. The more curvature or deformation the response surface plot shows, the more impact the pairwise interactions have on the response.

All percentages of FA, LS, and MS are based on the total dry mass of the soil. Runs 15–17 represent center-point replicate tests used to estimate pure error. The original Box–Behnken design generated 15 design points plus 3 center-point replicates; one center-point replicate was excluded due to specimen preparation deviation, resulting in 17 effective runs. Unconfined compressive strength test results were shown in Table 7.

In response surface models, the basis for judging the strength of interaction between factors are contour line shape and degree of surface deformation in 3D surface plots. Elliptical contours and high degree of surface deformation represent a strong interaction between factors, while circular contours and gentle slopes represent weak interaction between factors. Parallel straight contours and inclined planes represent no interaction between factors.

When the magnesium slag content was 7 wt.%, the contour line shape was elliptical and densely clustered contours representing strength response intervals were present. The major axis of the ellipse was not perpendicular or parallel to the coordinate axes. The corresponding 3D surface plot showed obvious deformation, and the gradient between high and low strength was pronounced. A strength peak of approximately 0.9 MPa was present in the range of 15–20 wt.% fly ash and 10–13 wt.% lithium slag. The contour line shape was elliptical and approached concentric circles. The contour of circular strength response intervals was sparsely distributed, and the boundary gradually decreased. The strength gradient difference in the 3D surface plot was relatively flat, and the gradient difference was much smaller than that of the fly ash–lithium slag combination. A strength peak of approximately 0.85 MPa was present only in the range of 15–18 wt.% fly ash and 6–8 wt.% magnesium slag. These characteristics indicate that there was a strong interaction between fly ash and lithium slag. The proportional synergy of this combination mainly affects the 7-day strength; if the ratio is unbalanced, the strength will drop dramatically. Based on the above analysis, the optimal proportion interval was determined to be 15.926 wt.% fly ash and 10.158 wt.% lithium slag. Subsequent verification results demonstrated that, keeping the lithium slag content at 10 wt.%, compressive strength rose considerably as fly ash dosage increased. When the fly ash content exceeded 20 wt.%, however, strength declined markedly. This outcome is consistent with the significant effect of fly ash observed in the ANOVA and with the quadratic curvature inherent in the quadratic model.

When the lithium slag content was fixed at 10 wt.%, the contour line shape was weakly elliptical and approached concentric circles. The contour of circular strength response intervals was sparsely distributed. The boundary gradually decreased. The 3D surface plot was relatively flat, and the strength gradient difference was much smaller than that of the fly ash–lithium slag combination. A strength peak of approximately 0.85 MPa was present only in the range of 15–18 wt.% fly ash and 6–8 wt.% magnesium slag. These results indicated that there was a weak interaction between fly ash and magnesium slag. In this combination, the effect of fly ash on strength is much greater than that of magnesium slag. When the magnesium slag content varied across the full 0–13 wt.% range, the variation in strength was less than 0.1 MPa. Therefore, the regulatory effect of magnesium slag was very weak and failed to significantly improve the 7-day strength. This result is completely consistent with the ANOVA results. The result showed that the effect of magnesium slag was not statistically significant.

When the fly ash content was fixed at 10 wt.%, the contour lines nearly formed a circle. The boundary of the interval of strength was unclear, and the contour was extremely sparse. There was almost no region with dense clustering. The deformations of the 3D surface plot were almost none, and it was nearly a uniformly sloping plane. The strength was constantly in the interval of 0.6–0.8 MPa, without any peak or trough. This means that the interaction between lithium slag and magnesium slag was extremely weak.

In this combination, the variation in the content of lithium slag and magnesium slag had no significant effect on the 7-day strength. No matter what the ratio between lithium slag and magnesium slag was adjusted to, the strength was always at a low level. This is totally consistent with the ANOVA result that the impact of lithium slag was not significant.

When the content of lithium slag was fixed at 7 wt.%, the contour lines nearly formed a parallel linear pattern, and there was neither any elliptical feature nor a circular feature. The distance between two adjacent contour lines was uniform, and they were linearly distributed along the fly ash direction. The corresponding 3D surface plot nearly formed a uniformly sloping plane with negligible deformations. The compressive strength increased linearly with the increase in lithium slag and decreased linearly with the increase in magnesium slag, and there was no peak of strength. This means that there was no interaction between fly ash and magnesium slag in this combination. In this combination, the effects of fly ash and magnesium slag were mutually independent, and there was no synergistic or antagonistic interplay between them. That is, when the content of fly ash increased by 5 wt.%, the strength increased by 0.10–0.15 MPa; and when the content of magnesium slag increased by 5 wt.%, the strength decreased by 0.08–0.10 MPa. This was consistent with the ANOVA result that the impact of magnesium slag approached the significance level, but it seemed to be contrary to the ANOVA result that the impact of lithium slag was not significant. This may be caused by the fact that the goodness-of-fit of Linear model was not so high, and it may not be sensitive enough to reflect the impact of lithium slag.

When the magnesium slag content was 7 wt.%, the contour line presented a weak nearly parallel linear form with uneven spacing and no elliptic phenomenon. In addition, the 3D surface plot was slightly inclined, but there was no obvious deformation. A relatively high strength value of approximately 1.5 MPa was achieved with a lithium slag content near 10 wt.% and a fly ash content around 15 wt.%. The aforementioned results imply a weak interaction between fly ash and lithium slag. Within this combination, the effect of lithium slag on strength was distinctly positive, whereas that of fly ash was comparatively weak. When the lithium slag dosage was increased from 0 wt.% to 10 wt.%, the strength rose from 1.0 MPa to 1.5 MPa, displaying a clear linear trend. These findings are entirely consistent with the ANOVA results, which identified the influence of lithium slag on strength as statistically significant.

When the fly ash content was 13 wt.%, the contour line presented a strictly parallel linear form with uniform spacing. The 3D surface plot appeared as a regular inclined plane with no deformation. The compressive strength was the independent superposition of positive effect of lithium slag and negative effect of magnesium slag with no interactive interference. It indicated that there was no interaction between lithium slag and magnesium slag. In this combination, lithium slag played a role in improving strength, and magnesium slag had a negative effect on strength. Because the above two effects occurred independently and added up, the proportion of mixture should be maximized to 10 wt.% for lithium slag and minimized to 2 wt.% for magnesium slag. The above results were exactly consistent with the optimal proportion analysis results. In addition, it was also consistent with the ANOVA results. The influence of lithium slag on strength was statistically significant according to the ANOVA results, and the influence of magnesium slag on strength approached significance.

**Figure 6:**
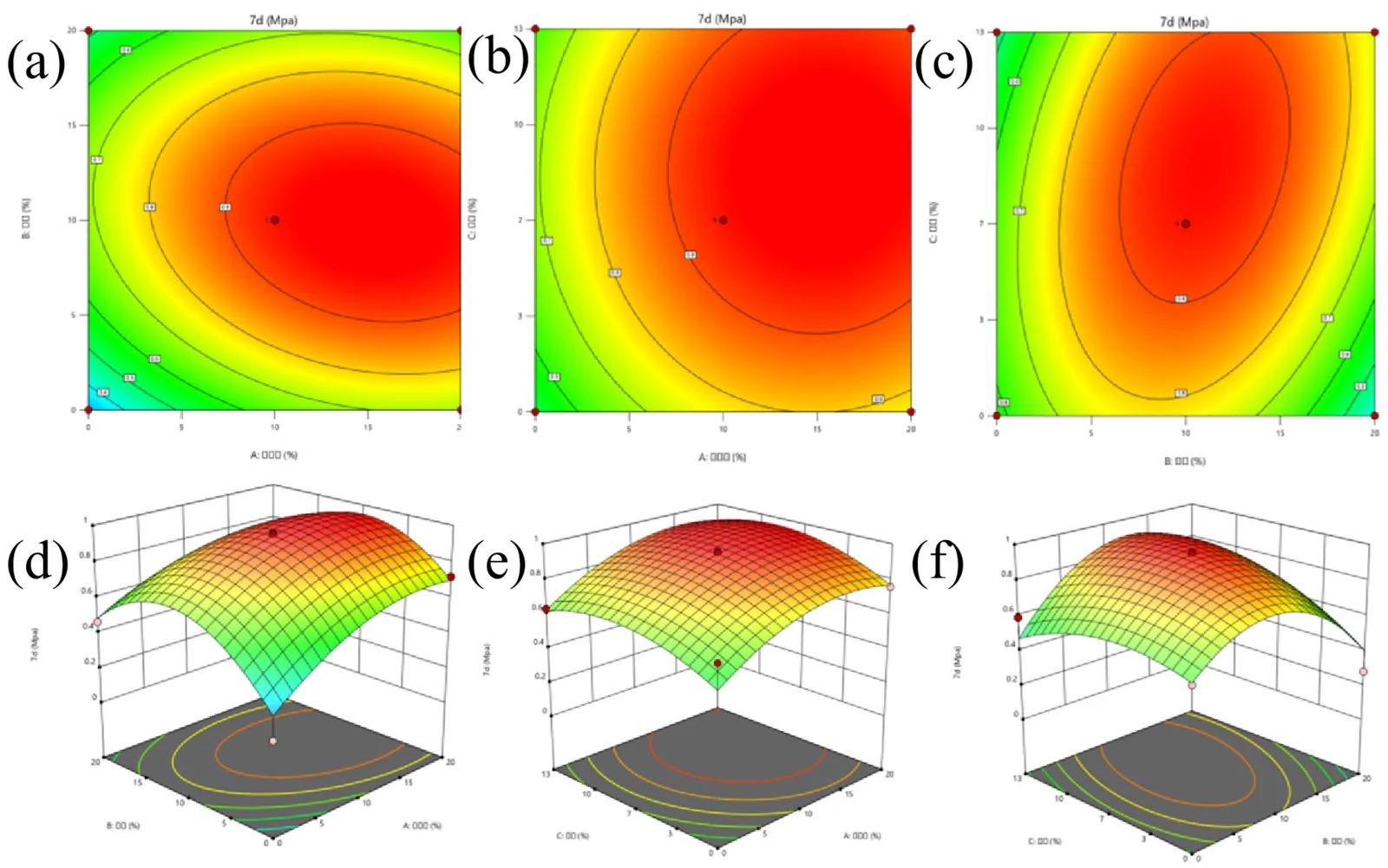
Unconfined compressive strength response surfaces at 7-day curing: (**a**) FA–LS contour plot; (**b**) FA–MS contour plot; (**c**) LS–MS contour plot; (**d**) FA–LS 3D response surface; (**e**) FA–MS 3D response surface; (**f**) LS–MS 3D response surface.

**Figure 7:**
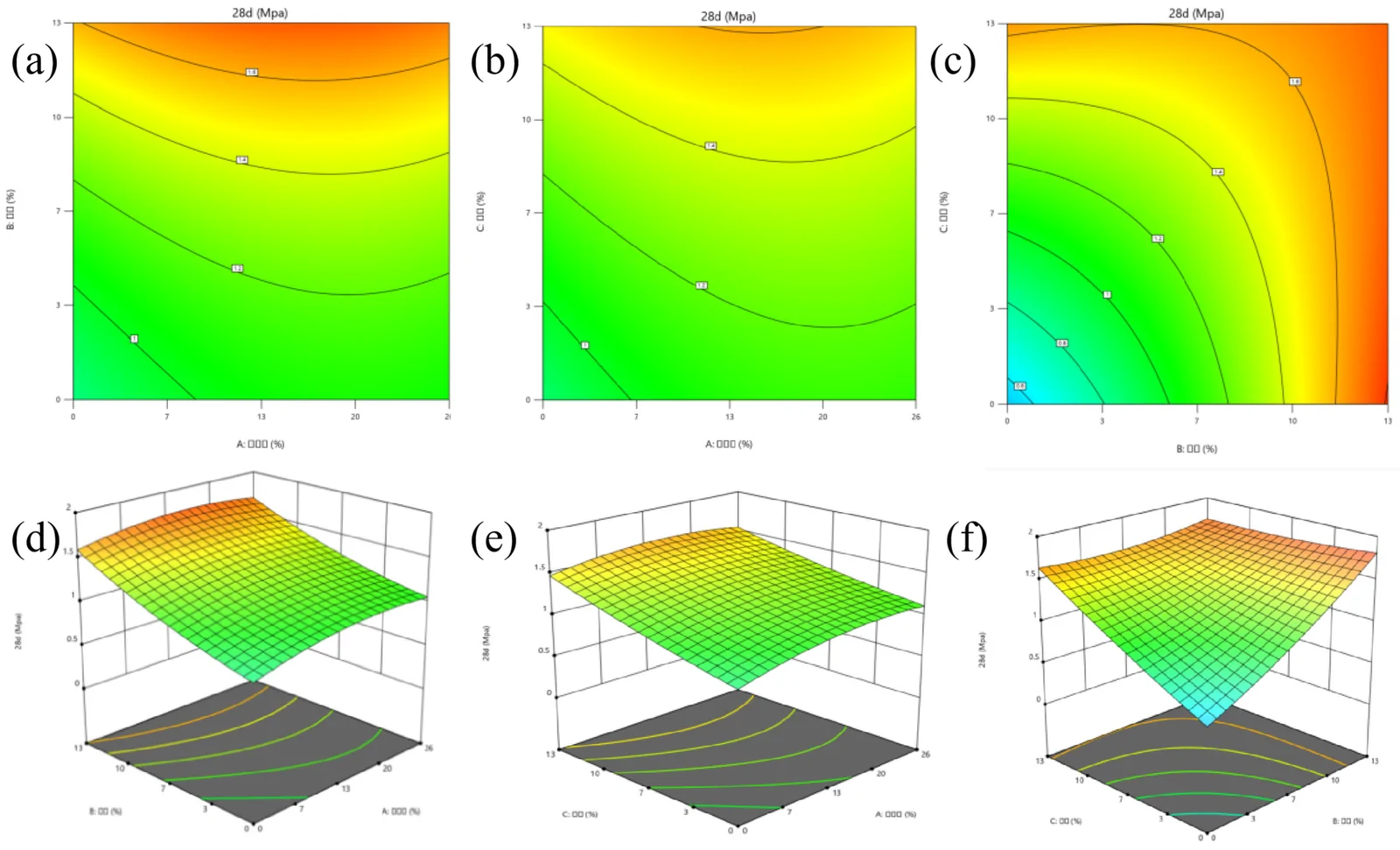
Unconfined compressive strength response surfaces at 28-day curing: (**a**) FA–LS contour plot; (**b**) FA–MS contour plot; (**c**) LS–MS contour plot; (**d**) FA–LS 3D response surface; (**e**) FA–MS 3D response surface; (**f**) LS–MS 3D response surface.

Depicted in Figure 6 and Figure 7 are the contour and three-dimensional response surface plots for compressive strength at 7 days and 28 days of curing, respectively. The corresponding features and strength development mechanisms are substantially different between the 7-day and 28-day curing ages.

During the initial 7 days of curing, the cementitious system is at a primary hydration stage. Pozzolanic reactions generate hydration products such as calcium silicate hydrate (C-S-H) gel—an amorphous binding phase that fills pores and cements soil particles together—and calcium aluminate hydrates, whose cementation provides the main source of compressive strength. Differences in strength values arise largely from the inherent reactivity of individual admixtures and the synergistic interplay among them.

The reactive part of fly ash is mostly vitreous SiO_2_ and Al_2_O_3_, and the reactive part of lithium slag is Li_2_O and highly reactive vitreous phases. When the two materials are blended together, the reactive lithium slag acts as a catalyst for hydration reactions. The spherical fly ash particles break through the vitreous network structure of fly ash particles and enter into the voids formed within lithium slag hydration products, disrupting the network structure of lithium slag hydration products. Hence, the dissolution of SiO_2_ and Al_2_O_3_ and their entry into the hydration reaction are facilitated. In addition, the hydration products derived from lithium slag act as a catalyst for the hydration of fly ash particles, and the spherical morphology of fly ash particles enables them to fill the pores in the lithium slag hydration products. The synergistic interaction of these two processes results in a “catalysis–filling” phenomenon. Through this interaction, a large volume of highly reactive C-S-H gel is produced.

An appropriate proportion range of fly ash, lithium slag, and magnesium slag that can reach a balance between reactivity excitation efficiency and particle packing is taken as the optimal proportion range, and the corresponding strength peak is obtained. Otherwise, if there is an imbalance between the proportions of these three types of slag, that is, insufficient lithium slag content to further excite the reactivity of fly ash or too high lithium slag content causing an increase in porosity of the system, the strength will decrease rapidly. This phenomenon is perfectly consistent with the significant deformation presented in the response surface plots in Figure 6 and Figure 7 and the fact that the contour lines present an obviously elliptical shape. This result also explains the significant influence of fly ash obtained in the analysis of variance.

The main reactive components of magnesium slag are MgO and CaO. The main products of its hydration reactions are magnesium hydroxide and calcium silicate hydrates, but its reaction rate is much slower than that of lithium slag. When the curing age reaches 7 d, the degree of hydration of magnesium slag is still relatively low, and then magnesium slag is unable to induce the pozzolanic reactivity of fly ash to be activated. The interaction between fly ash and magnesium slag is only a simple physical superposition effect during this period. Therefore, the strength development of the system is largely attributed to the reactive performance of fly ash, and the regulation effect of magnesium slag is limited. This phenomenon is consistent with the characteristics of response surface, namely, almost concentric circular contour lines and gentle slope of surface deformation. In addition, ANOVA result also indicates that the influence of magnesium slag is statistically unimportant.

The process of lithium slag and magnesium slag being activated are consuming Ca(OH)_2_ in the system. When lithium slag and magnesium slag are applied in one system, the competition for available calcium source occurs, and then the rate of pozzolanic reactions is limited. In addition, the particle size distribution of magnesium slag and lithium slag is not complementary, and cannot form an effective and densely packed microstructure. When the curing age is 7 d, the amount of hydration products generated is limited, and the distribution of hydration products is loose and not consolidated. Then, the compressive strength ranges in the limited value of 0.6–0.8 MPa, and the response surface does not exhibit any peak or trough. This phenomenon is consistent with the ANOVA result that the influence of lithium slag is not statistically significant.

When the curing age reaches 28 d, the rate of hydration reaction in the cementitious system tends to be relatively stable, and the rate of strength gain is limited. The type of hydration products and matrix microstructure are basically determined. At this time, the mode of action of the individual admixture changes from “synergistic activation” to “independent superposition of effects”. This is the essence why the 28-day compressive strength could be fit into a Linear model.

By this age, the hydration reaction of fly ash, lithium slag, and magnesium slag has approached to be nearly complete, and the effect of each admixture becomes individual. The cementitious strength originates from pozzolanic reaction products of fly ash; the gel matrix compactness is continuously enhanced by the catalytic effect of lithium slag, and the cementitious effect of magnesium slag proportion is inherently weakened due to the extremely low cementitiousness of magnesium hydroxide hydration products. Since no obvious synergistic or antagonistic effect emerged among the three components, the total strength is linearly superposed by the corresponding individual effects. The parallel linear contour lines and regular inclined planar surfaces shown in response surface plots are in agreement with this situation.

In this case, the ANOVA result that the influence of magnesium slag became statistically significant can be interpreted as the cumulative effect of its negative proportion at later curing stages. On the other hand, the lack of statistical significance of the influence of fly ash in ANOVA may be caused by the relatively low goodness-of-fit of the linear model, and the linear model is not sensitive enough to capture the subtle influence of fly ash at later hydration stages.

There is still some residual activity of catalytic activity of lithium slag, and it would further promote the secondary hydration of fly ash; however, since the porosity of the system has already been stabilized at this proportion, the total effect manifests as a weak interaction only. The strength shows a linear increase with the increase in the proportion of lithium slag, which is in full agreement with the result of ANOVA that the influence of lithium slag became statistically significant.

The 7-day and 28-day UCS models were statistically significant, with model F-values of 5.43 and 15.87, respectively. For the 7-day model, the lack of fit was statistically significant (F = 181.75, *p* < 0.0001), indicating that some variability remained unexplained by the fitted model. In contrast, the lack of fit of the 28-day linear model was not statistically significant (F = 3.00, *p* = 0.2731), indicating that the linear model adequately represented the experimental data within the investigated design space. For the 28-day model, lithium slag (B) and magnesium slag (C) were statistically significant factors, whereas fly ash (A) was not significant.

Results of response surface optimization design are shown in Table 8 and Table 9. The experimental results may suggest that the reliability analysis of the models provides a sufficient foundation for optimization analysis using the software to determine the optimal test conditions, the optimal parameters are shown in Table 10. Moreover, the significant findings could indicate that the optimal mix for 7-day compressive strength, designated as Mix a, appears to consist of 15.926 wt.% fly ash, 10.158 wt.% lithium slag, and 6.427 wt.% magnesium slag. In light of the key results derived with a high desirability value of 0.967, the evidence may suggest that the optimal mix for 28-day compressive strength, designated as Mix b, could demonstrate a composition of 16.064 wt.% fly ash, 10 wt.% lithium slag, and 2 wt.% magnesium slag. Furthermore, the results might indicate that under these optimal conditions, the mean unconfined compressive strength of the stabilized specimens appears to reach 0.974 MPa at 7 days and 1.834 MPa at 28 days. Model predictions match experimental values, confirming model reliability.

### 3.2. Dynamic Ultimate Deformation Analysis

**Figure 8:**
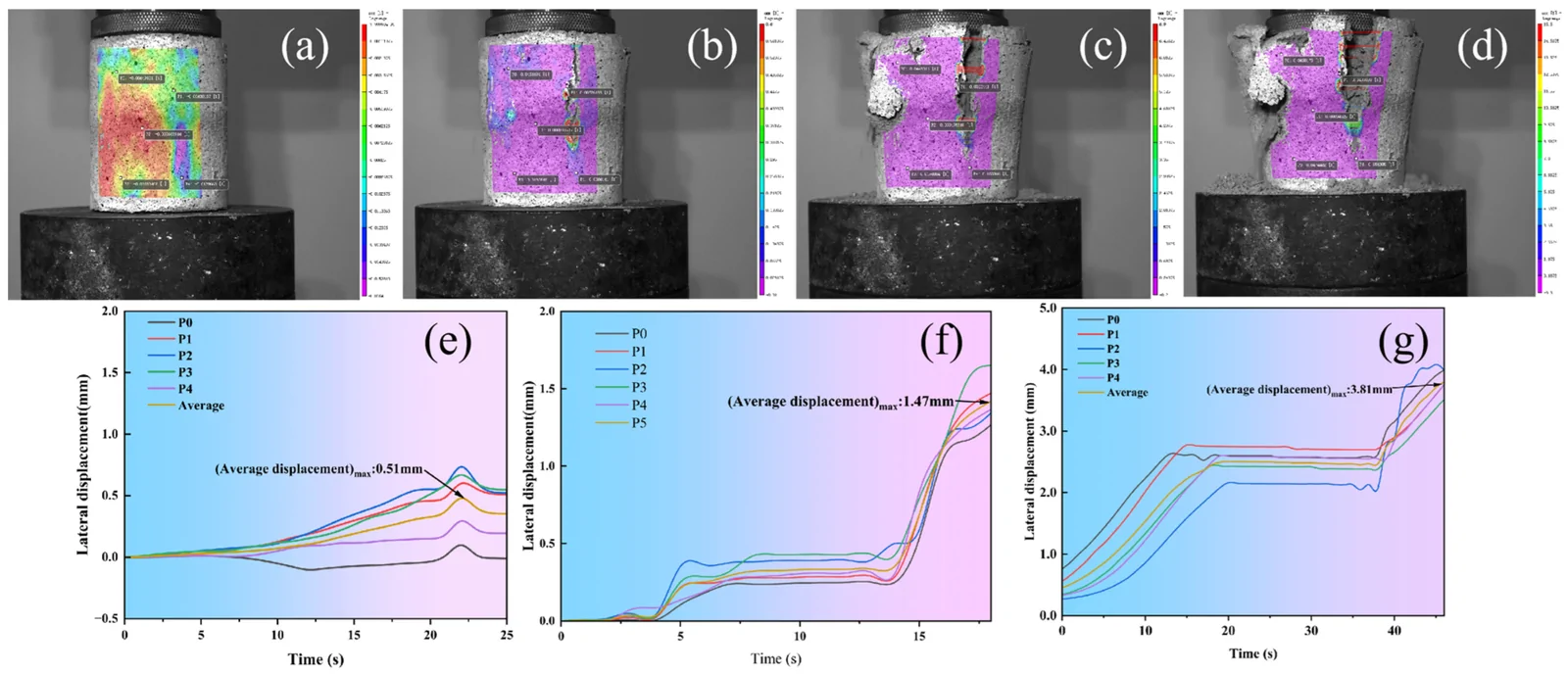
Dynamic ultimate deformation images: (**a**–**d**) displacement variation images; (**e**) transverse displacement curve of pure loess group; (**f**) transverse displacement curve of 7 d-Optimal Mix a; (**g**) transverse displacement curve of 28 d-Optimal Mix b.

Figure 8 presents the relevant three-dimensional deformation images and transverse displacement curves at selected data points for both unstabilized and stabilized loess specimens. Moreover, the significant findings may suggest that examining the deformation and cracking processes revealed in the 3D deformation images could demonstrate important patterns in crack propagation and damage morphology of the stabilized specimens. Furthermore, the key results might indicate that the displacement of randomly selected monitoring points throughout the entire loading process appears to support the effectiveness of stabilization. Additionally, the evidence could suggest that tracking these significant deformation patterns provides evidence that the methodology demonstrates reliable evaluation capacity across specimen types. Findings show crack behavior differs between groups.

However, the stabilized specimen corresponding to the 7-day Optimal Mix a exhibited distinct linear elastic behavior that may suggest a well-defined yielding stage during the loading process. Given that the evidence demonstrates localized deformation occurred upon exceeding the yield point, the significant results could indicate that structural failure followed a predictable progression [25,26]. Moreover, the findings might suggest that the maximum average transverse displacement for this specimen could demonstrate a value of +1.47 mm. In light of these key results, the evidence may indicate that the unstabilized pure loess specimen displayed a notably more gradual deformation progression that appears to reflect uniform loading response throughout the entire loading sequence. Results show the stabilized specimen fails faster. Nevertheless, the significant findings may suggest that the time to structural failure for the stabilized specimen could demonstrate prolonged resistance, reaching up to 47 s, while the evidence might indicate that the average maximum transverse displacement appears limited to +3.82 mm. Furthermore, the key results could suggest that the substantial disparity in ultimate transverse displacement between the two groups may demonstrate that the 28-day Optimal Mix b shows that the cementation effect appears pronounced. Notwithstanding the observed displacement differences, the significant evidence might indicate that superior resistance to dispersed cracking could demonstrate enhanced structural integrity in the stabilized loess specimens. Therefore, the findings may suggest that the degree of disintegration upon failure appears considerably milder, which could indicate that the results demonstrate delayed structural collapse and enhanced resistance to damage [27,28]. Data shows stabilized loess resists collapse better.

### 3.3. XRD Phase Analysis

**Figure 9:**
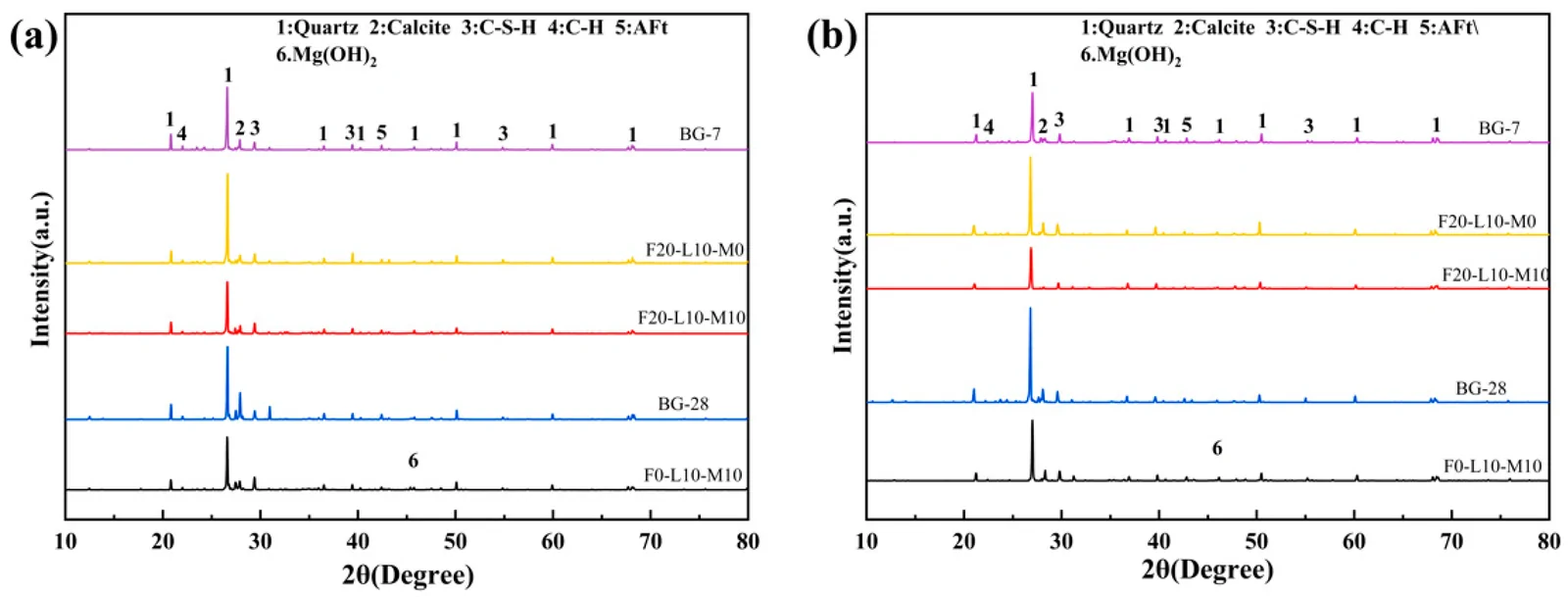
XRD patterns of stabilized loess groups: (**a**) 7-day curing; (**b**) 28-day curing.

When the NaOH activator concentration was fixed at 5 wt.%, representative specimens with different proportions of cementitious phases were selected for XRD analysis after 7 and 28 days of curing. The results are presented in Figure 9. The curing period significantly affected the phase evolution of the stabilized specimens, which is consistent with the variation in UCS results discussed previously.

As shown in Figure 9a, all specimens cured for 7 days exhibited a broad and diffuse diffraction feature around 2θ = 30°. This feature is generally associated with poorly crystalline or amorphous hydration products formed during alkali activation. Compared with other groups, the diffuse feature was relatively weak in Group 8 and reached the lowest intensity in Group 7, whereas Optimal Mix a exhibited a more pronounced diffuse feature. This difference may be attributed to the combined effects of the physical filling effect of fly ash and the occurrence of early-age pozzolanic reactions, which could promote the formation of additional poorly crystalline cementitious products.

In Group 7, the absence of fly ash reduced the availability of reactive aluminosilicate sources, which may have limited the formation of aluminosilicate-related hydration products during the early curing stage. Diffraction peaks corresponding to ettringite (AFt) were observed in most groups, indicating that aluminum- and sulfate-related reactions occurred during early hydration. However, no obvious AFt peaks were detected in Group 7, which may be related to the insufficient supply of reactive aluminum sources due to the absence of fly ash.

Diffraction peaks attributed to Ca(OH)_2_ were also observed in all groups. The variation in Ca(OH)_2_ peak intensity reflects the balance between its generation and consumption during hydration and pozzolanic reactions. Group 7 exhibited the strongest Ca(OH)_2_ diffraction peak intensity and also showed additional peaks possibly attributed to Mg(OH)_2_, which may be related to the hydration reactions of magnesium slag. These results suggest enhanced hydration activity of magnesium slag in this group.

Compared with Optimal Mix a, the Ca(OH)_2_ diffraction peaks in Group 4 were relatively weak. Since magnesium slag was absent in this group, the additional Ca-containing hydration products generated from magnesium slag hydration were unavailable. Meanwhile, the Ca(OH)_2_ produced under alkaline activation conditions may have been continuously consumed by pozzolanic reactions involving reactive components from fly ash and lithium slag. Therefore, the relatively low Ca(OH)_2_ intensity in Group 4 suggests active secondary reactions and enhanced formation of poorly crystalline hydration products.

Compared with Group 8, the magnesium slag content in Optimal Mix a was slightly lower at 7 days. This reduced the possibility of excessive Ca-containing phase accumulation and promoted a more balanced interaction among magnesium slag, fly ash, and lithium slag during early hydration.

For the specimens cured for 28 days, the phase evolution of each group is shown in Figure 9b. Compared with the 7-day specimens, the intensity of Ca(OH)_2_ diffraction peaks decreased significantly in most groups, and some peaks became difficult to distinguish. Ca(OH)_2_ participates in secondary pozzolanic reactions with reactive silica and alumina phases from fly ash and lithium slag; therefore, its reduced diffraction intensity with increasing curing age suggests its progressive consumption during hydration.

However, detectable Ca(OH)_2_ peaks remained in Group 4, indicating that part of the Ca-containing phase was retained after 28 days of curing. With increasing curing age, the diffuse diffraction feature associated with poorly crystalline hydration products became more pronounced in all groups, especially in specimens containing both fly ash and lithium slag. This observation suggests that continuous formation of low-crystallinity hydration products occurred during long-term curing.

For groups containing magnesium slag, the gradual reduction in Ca(OH)_2_ diffraction intensity from 7 to 28 days indicates that Ca-containing phases were involved in ongoing secondary reactions. In contrast, groups without magnesium slag showed relatively weak Ca(OH)_2_ diffraction peaks throughout the curing period.

It should be noted that the diffuse XRD features observed in this study cannot independently provide definitive identification or quantitative analysis of C-(A)-S-H phases. Therefore, the possible formation of C-(A)-S-H-type products and other hydration products discussed above is proposed based on the combined evidence from XRD characteristics, raw material composition, alkaline activation conditions, and previous studies. Further techniques such as SEM-EDS, FTIR, or NMR would be required for more accurate phase identification.

### 3.4. Microstructural Analysis by SEM

**Figure 10:**
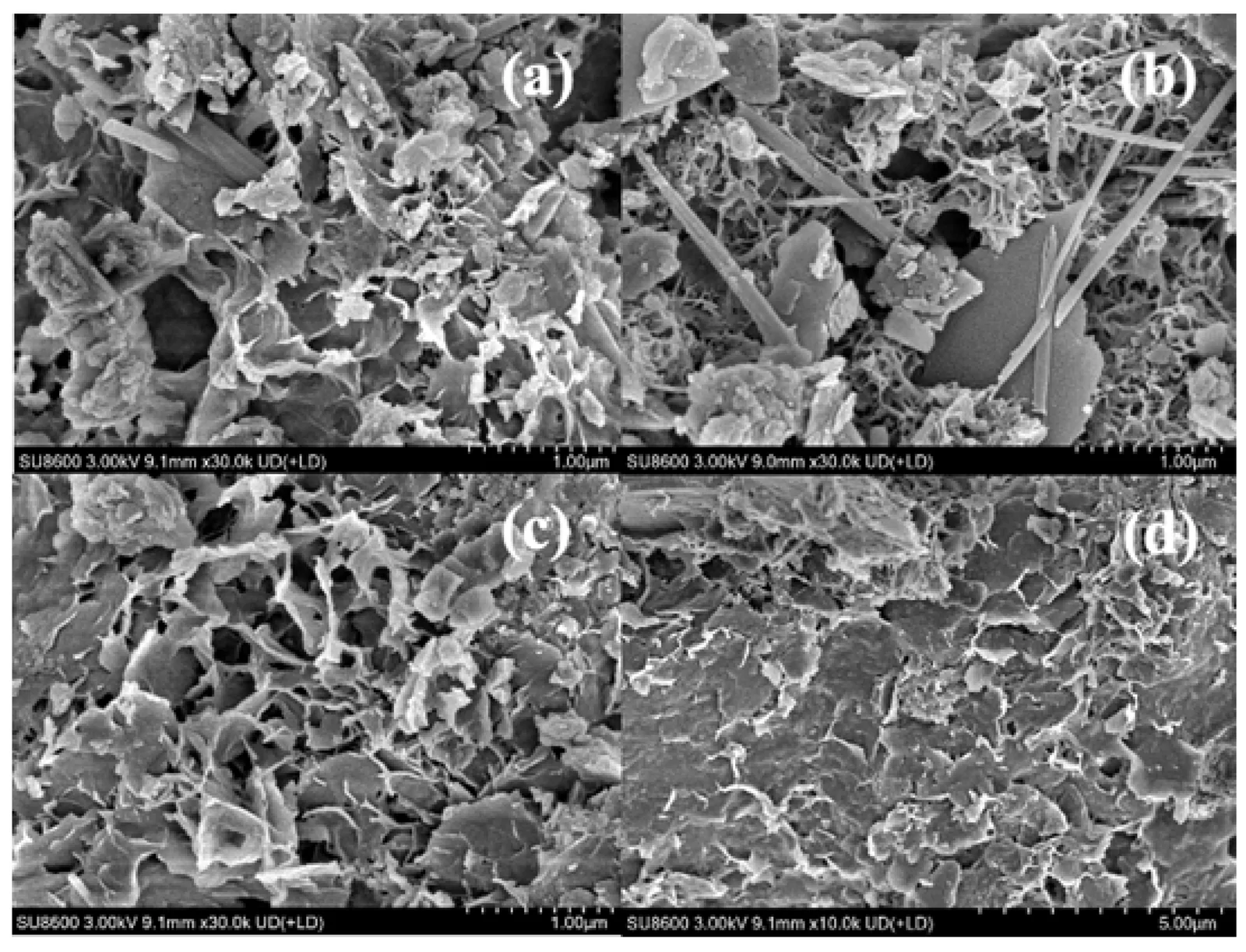
SEM images of stabilized loess groups at 7-day curing age: (**a**) 28 d-Optimal Mix b; (**b**) 7 d-Optimal Mix a; (**c**) Group 4 (F20-L10-M0); (**d**) Group 7 (F0-L10-M10).

**Figure 11:**
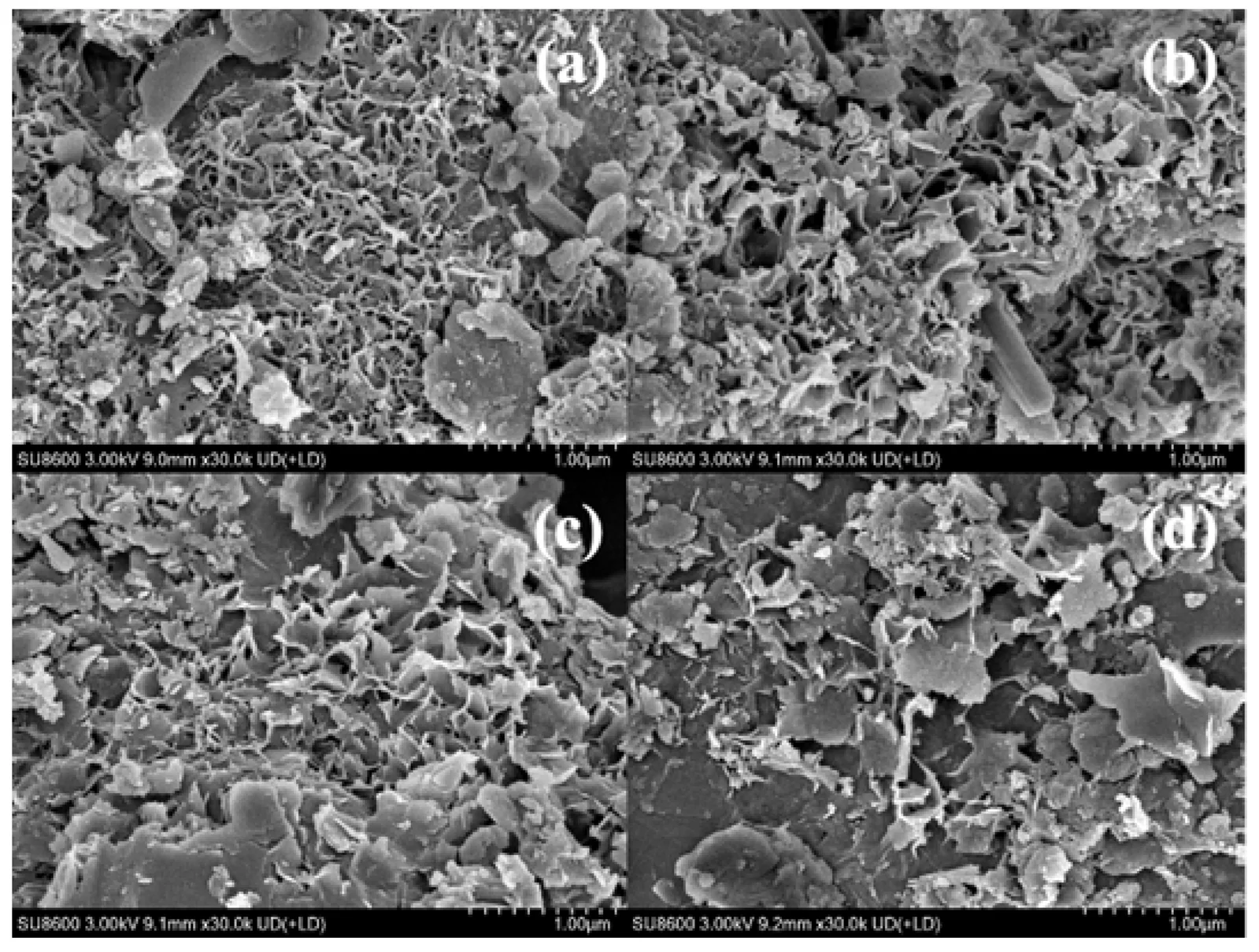
SEM images of stabilized loess groups at 28-day curing age: (**a**) 28 d-Optimal Mix b; (**b**) 7 d-Optimal Mix a; (**c**) Group 4 (F20-L10-M0); (**d**) Group 7 (F0-L10-M10).

Selected groups of specimens were examined using scanning electron microscopy (SEM) in conjunction with XRD analysis to investigate the microstructural evolution of stabilized loess during the curing period, as illustrated in Figure 10 and Figure 11.

From the SEM images of specimens cured for 7 days (Figure 10a), it can be observed that a continuous reaction product network gradually developed between soil particles, although numerous pores remained within the matrix. A large amount of flocculent and gel-like products was observed covering the surfaces of soil particles and filling the surrounding pores. These gel-like structures are considered to be associated with the formation of poorly crystalline hydration products during alkaline activation. Considering the coexistence of Ca-, Si-, and Al-containing reactive components in the system, the formation of C-(A)-S-H-type products is proposed. However, SEM morphology alone cannot provide definitive phase identification.

Needle-like crystals were also observed within the gel matrix, which may correspond to ettringite-related products based on the corresponding XRD results. In addition, plate-like crystals were observed throughout the matrix. Combined with the XRD analysis, these crystals may be related to Ca-containing crystalline phases, such as Ca(OH)_2_. The presence of these crystalline products suggests that hydration and pozzolanic reactions occurred during the early curing stage. The catalytic effect of lithium slag may contribute to the continuous reaction process, especially in the absence of magnesium slag.

For the specimens cured for 28 days (Figure 11a,b), Optimal Mix b exhibited a denser and better-connected microstructure, with fewer visible pores compared with the 7-day specimens. The previously observed plate-like crystalline structures became less apparent, suggesting the consumption or transformation of some crystalline phases during prolonged curing. Some needle-like structures remained embedded in the gel matrix, while the particle interfaces became increasingly filled with continuous reaction products, resulting in a more compact hardened structure. Based on previous studies and the XRD results, the formation of AFm-type products is considered possible; however, their specific identification requires further elemental characterization.

For Optimal Mix a at 28 days (Figure 11a,b), locally distributed plate-like and loosely flocculent structures were observed within the matrix. These morphologies may be related to magnesium-containing hydration products, such as brucite or magnesium silicate hydrate (M-S-H)-type products, based on the presence of magnesium slag and previous studies. However, without SEM-EDS analysis, these phases cannot be conclusively identified solely from morphology. The formation of these magnesium-related products may influence the pore structure and local compactness of the matrix, and excessive expansion associated with magnesium slag hydration could potentially contribute to microstructural defects.

Comparison of Figure 10c,d and Figure 11c,d shows that although the specimen without magnesium slag developed a relatively compact microstructure after curing, its mechanical strength remained limited. Compared with the optimal mixtures, the positive contribution of magnesium slag to the activation environment and reaction process is suggested. In addition, the specimen without fly ash exhibited a relatively loose microstructure. Although magnesium-containing reaction products may partially fill the pores, the overall cementation ability remained insufficient due to the lack of reactive aluminosilicate sources from fly ash. Therefore, fly ash appears to play an important role in providing reactive components and promoting the formation of the interconnected cementitious matrix in the ternary system.

Compared with Optimal Mix a at 7 days (Figure 10b), the 28-day microstructure exhibited further development of reaction products and improved particle bonding. In Optimal Mix a, a more developed gel-like matrix and longer needle-like crystals were observed, while fewer plate-like crystalline products were visible. These observations suggest enhanced hydration and pozzolanic reactions in the ternary system, which may contribute to the improved compressive strength.

In summary, the optimal mixes exhibit the highest compressive strength because they achieve a precise balance between chemical activation efficiency and physical microstructural packing. For the 7-day Optimal Mix a, the specific proportion of magnesium slag supplies an optimal alkaline environment that maximizes the early-age “catalysis-filling” synergistic effect between fly ash and lithium slag, thereby generating the highest yield of C-S-H gel and ettringite to bind the soil particles without causing Ca(OH)_2_ accumulation. Conversely, for the 28-day Optimal Mix b, the significantly reduced magnesium slag content prevents the formation of late-stage micro-defects and microcracking caused by the expansion of magnesium-based hydration products. Coupled with the sustained pozzolanic reactions of fly ash and the continuous catalytic effect of lithium slag, this optimal ratio facilitates the development of a highly dense, continuous, and defect-free cementitious network.

## 4. Conclusions

The findings of this study demonstrate the potential of using fly ash, lithium slag, and magnesium slag as sustainable materials for loess stabilization. The proposed ternary solid waste system provides an effective approach for improving loess engineering performance while promoting the resource utilization of industrial by-products, which may contribute to the development of greener soil stabilization technologies:

1. A ternary fly ash–lithium slag–magnesium slag binder may suggest that collapsible loess from Northwest China could be effectively stabilized through combined binder action. However, the mix design could demonstrate that response surface methodology provides significant optimization support, and the key mechanical properties might indicate that UCS, DIC, XRD, and SEM evidence establishes important mechanistic findings.
2. The significant results may suggest that the optimal 7-day mix (15.926 wt.% fly ash, 10.158 wt.% lithium slag, and 6.427 wt.% magnesium slag) could demonstrate that 0.974 MPa strength appears attainable, while the 28-day optimum (16.064 wt.% fly ash, 10 wt.% lithium slag, and 2 wt.% magnesium slag) might indicate that 1.834 MPa results are achievable.
3. Strength evolution shifts from an early synergistic quadratic model to a linear model of independent contributions. XRD confirms that alkaline activation produces C-S-H gel and ettringite. Magnesium slag plays a critical role by supplying the alkaline environment, as evidenced by progressive Ca(OH)_2_ consumption.
4. SEM analysis indicates that pore filling plus chemical cementation is the mechanism responsible for microstructure evolution in the key mixes. Dense C S H and ettringite networks are observed in optimal mixes, whereas porous and poorly cemented structures are observed in fly ash-/or magnesium slag-deficient mixes. Fly ash acts as a reactive skeleton, and lithium slag efficiently promotes early hydration.
5. Magnesium slag is responsible for the regulation of alkalinity, and the synergistic effect of this regulation significantly enhances loess strength. Therefore, this is an effective and economical approach to the utilization of industrial solid waste.

Although this study clarifies the strengthening mechanism of the fly ash–lithium slag–magnesium slag stabilized loess system, the investigation was mainly based on laboratory tests. Further studies are needed to evaluate its long-term durability under complex environmental conditions and verify its performance through field applications.

## Figures and Tables

**Table 1:** Physical property baseline of the loess.

Material	Liquid Limit Value (%)	Plastic Limit Value (%)	Optimum Dry Density (g/cm^3^)	Optimum Water Content (%)
Loess	31.6	19.4	1.69	13.4

**Table 2:** Chemical compositions and physical properties of the stabilizing materials.

Property	Fly Ash (FA)	Lithium Slag (LS)	Magnesium Slag (MS)
Source	Class F fly ash collected from a coal-fired thermal power plant in Shaanxi Province, China	Lithium slag supplied by a lithium carbonate production plant in Jiangxi Province, China	Magnesium slag obtained from a magnesium smelting plant in Ningxia Hui Autonomous Region, China
SiO_2_ (wt.%)	54.82	47.36	28.64
Al_2_O_3_ (wt.%)	27.41	18.72	5.38
CaO (wt.%)	5.26	16.84	45.73
Fe_2_O_3_ (wt.%)	6.17	2.43	1.68
MgO (wt.%)	1.54	2.86	11.82
SO_3_ (wt.%)	0.61	3.54	1.24
K_2_O + Na_2_O (wt.%)	2.46	2.31	1.16
TiO_2_ (wt.%)	1.21	0.42	0.18
Loss on ignition (LOI, wt.%)	2.15	3.62	1.83
Specific surface area (Blaine, m^2^/kg)	392	468	445

**Table 3:** Goodness-of-fit analysis for compressive strength models.

Source	Sequential *p*-Value	Lack of Fit *p*-Value	Adjusted R^2^	Predicted R^2^	
Linear	0.3882	<0.0001	0.0165	−0.3217	
2FI	0.7969	<0.0001	−0.1601	−1.2664	
Quadratic	0.0012	0.0008	0.8512	0.6205	Suggested
Cubic	<0.0001		0.9964		Aliased

**Table 4:** Goodness-of-fit analysis for 28-day compressive strength models.

Source	Sequential *p*-Value	Lack of Fit *p*-Value	Adjusted R^2^	Predicted R^2^	
Linear	0.0004	0.2731	0.6081	0.3802	Suggested
2FI	0.1876	0.0007	0.6620	0.0425	
Quadratic	0.8584	0.0003	0.5915	−2.0508	
Cubic	0.0003		0.9834		Aliased

**Table 5:** Statistical variance analysis for 7-day compressive strength results.

Source	Sum of Squares	df	Mean Square	F-Value	*p*-Value	
Model	0.7302	9	0.0811	5.43	0.0181	significant
A—FA	0.1365	1	0.1365	9.14	0.0193	
B—LS	0.0044	1	0.0044	0.2959	0.6034	
C—MS	0.0268	1	0.0268	1.79	0.2222	
AB	0.0138	1	0.0138	0.9246	0.3683	
AC	0.0002	1	0.0002	0.0151	0.9058	
BC	0.0477	1	0.0477	3.20	0.1169	
A^2^	0.0679	1	0.0679	4.54	0.0705	
B^2^	0.3377	1	0.3377	22.62	0.0021	
C^2^	0.0537	1	0.0537	3.60	0.0997	
Residual	0.1045	7	0.0149			
Lack of Fit	0.1038	3	0.0346	181.75	<0.0001	significant
Pure Error	0.0008	4	0.0002			
Cor Total	0.8347	16				

**Table 6:** Statistical variance analysis for 28-day compressive strength results.

Source	Sum of Squares	df	Mean Square	F-Value	*p*-Value	
Model	0.7638	3	0.2549	15.87	0.0004	significant
A—FA	0.0245	1	0.0245	1.53	0.2432	
B—LS	0.4637	1	0.4637	28.88	0.0003	
C—MS	0.2764	1	0.2764	17.21	0.0018	
Residual	0.2087	13	0.0161			
Lack of Fit	0.1968	11	0.0179	3.00	0.2731	Not significant
Pure Error	0.0119	2	0.0060			
Cor Total	0.9735	16				

**Table 7:** Unconfined compressive strength test results (Box–Behnken design matrix; 17 effective runs, including 3 center-point replicates).

Run	FA/wt.%	LS/wt.%	MS/wt.%	7 d/MPa	28 d/MPa
1	0	0	0	0.15	0.2
2	20	0	0	0.37	0.67
3	0	10	0	0.62	1.34
4	20	10	0	0.75	1.54
5	0	0	10	0.56	1.21
6	20	0	10	0.83	1.63
7	0	10	10	0.99	1.60
8	20	10	10	1.21	1.89
9	0	5	5	0.40	0.87
10	26	5	5	0.45	0.95
11	10	0	5	0.34	0.69
12	10	13	5	0.67	1.44
13	10	5	3	0.55	1.18
14	10	5	13	0.65	1.21
15	10	5	5	0.54	1.11
16	10	5	5	0.57	1.20
17	10	5	5	0.49	1.09

**Table 8:** Results of 7-day response surface optimization design.

Number	FA	LS	MS	7 d	Desirability
1	15.926	10.158	6.427	0.974	1.000
2	16.914	9.136	9.453	0.972	1.000
3	12.979	9.107	6.834	0.970	1.000
4	15.040	12.672	8.292	0.971	1.000
5	16.365	10.775	10.224	0.975	1.000
6	17.579	10.787	6.404	0.964	1.000
7	16.222	11.778	8.378	0.978	1.000
8	15.679	9.259	6.885	0.978	1.000
9	18.074	9.309	6.709	0.967	1.000
10	16.318	9.189	6.954	0.977	1.000
11	12.444	9.556	9.389	0.968	1.000
12	17.406	10.173	9.536	0.975	1.000
13	13.073	9.122	6.102	0.964	1.000
14	14.034	13.492	10.119	0.960	1.000
15	18.124	9.536	8.826	0.972	1.000
16	13.450	12.279	8.302	0.973	1.000
17	16.529	11.772	7.636	0.973	1.000

**Table 9:** Results of 28-day response surface optimization design.

Number	FA	LS	MS	28 d	Desirability
1	16.064	10.000	2.000	1.834	0.967
2	18.947	10.000	2.001	1.834	0.967
3	19.344	10.000	2.000	1.834	0.967
4	19.579	13.000	2.000	1.834	0.967
5	18.366	13.000	2.000	1.834	0.967
6	18.804	12.997	2.000	1.834	0.967
7	17.986	13.000	2.000	1.834	0.966
8	17.738	13.000	2.000	1.833	0.966
9	20.450	13.000	2.000	1.833	0.966
10	18.170	13.000	2.036	1.833	0.966
11	19.159	12.989	2.000	1.833	0.966
12	20.997	13.000	2.000	1.832	0.965
13	16.732	13.000	2.000	1.831	0.965
14	21.590	13.000	2.000	1.831	0.964
15	20.557	13.000	2.163	1.830	0.963
16	19.896	12.951	2.000	1.828	0.963
17	15.660	13.000	2.000	1.827	0.963

**Table 10:** Optimal parameters.

Mix Designation	Fly Ash Content (wt.%)	Magnesium Slag Content (wt.%)	Lithium Slag Content (wt.%)	Compressive Strength (MPa)
7 d-Optimal Mix a	15.926	6.427	10.158	0.974
28 d-Optimal Mix b	16.064	2	10	1.834

## Data Availability

The original contributions presented in this study are included in the article. Further inquiries can be directed to the corresponding authors.
